# Correction to *Journal of Cosmetic Dermatology* Articles

**DOI:** 10.1111/jocd.71162

**Published:** 2026-10-08

B. S. F. Bravo , T. Calvacante , C. S. Nobre , L. G. Bravo , M. C. Zafra , and M. Calomeni , “Exploring the Safety and Satisfaction of Patients Injected With Collagen Biostimulators—A Prospective Investigation Into Injectable Poly‐l‐Lactic Acid (PLLA),” Journal of Cosmetic Dermatology 24 (2025): e16723, 10.1111/jocd.16723.PMC1184595239663956

B. S. F. Bravo , R. D. M. Carvalho , M. Calomeni , et al., “Nasal Filling Guided by High Frequency Ultrasound: Reducing Risks,” Journal of Cosmetic Dermatology 23 (2024): 3256–3262, 10.1111/jocd.16430.PMC1364908738958454

B. S. F. Bravo , R. de Melo Carvalho , L. Penedo , et al., “Applied Anatomy of the Layers and Soft Tissues of the Forehead During Minimally‐Invasive Aesthetic Procedures,” Journal of Cosmetic Dermatology 21 (2022): 5864–5871, 10.1111/jocd.15131.35634970

B. S. F. Bravo , T. Cavalcante , C. Silveira , L. G. Bravo , M. C. Zafra , and M. Calomeni , “Resolve and Dissolve—An Ultrasound‐Guided Investigation on the Effects of Hyaluronidase on Different Soft Tissue Fillers,” Journal of Cosmetic Dermatology 23 (2024): 3173–3181, 10.1111/jocd.16393.PMC1364908338769647

B. S. F. Bravo , M. Calomeni , C. R. M. da Rocha , and R. D. M. Carvalho , “Supraperiosteal Application of Hyaluronic Acid With Cannula: Is It Possible?,” Journal of Cosmetic Dermatology 20 (2021): 3178–3181, 10.1111/jocd.14241.34021961

The format of Mariana Calomeni's name has been corrected in the above‐mentioned articles.

We apologize for this error.

